# Ninjinyoeito Prevents Onset of Depression-Like Behavior and Reduces Hippocampal iNOS Expression in Senescence-Accelerated Mouse Prone 8 Mice

**DOI:** 10.1155/2023/2151004

**Published:** 2023-08-09

**Authors:** Chise Taniguchi, Takuya Watanabe, Marika Hirata, Akinobu Hatae, Kaori Kubota, Shutaro Katsurabayashi, Katsunori Iwasaki

**Affiliations:** Department of Neuropharmacology, Faculty of Pharmaceutical Sciences, Fukuoka University, 8-19-1 Nanakuma, Jonan-ku, Fukuoka 814-0180, Japan

## Abstract

Late-life depression is a globally prevalent disorder. Ninjinyoeito (NYT), a traditional Japanese herbal medicine, attenuates depressive symptoms in older patients. However, the mechanisms underlying the antidepressive effect of NYT are unknown. In this study, we investigated the mechanism of the action of NYT using senescence-accelerated mouse prone 8 (SAMP8) mice, which exhibit accelerated aging. SAMP8 mice were treated with NYT starting at 12 weeks of age. Twelve-week-old SAMP8 mice did not show prolonged immobility time in the tail suspension test compared with age-matched SAMR1 mice (normal aging control). At 34 weeks of age, vehicle-treated SAMP8 mice displayed prolonged immobility time compared with SAMR1 mice. NYT-treated SAMP8 mice showed a shorter immobility time than that of vehicle-treated SAMP8 mice. Notably, NYT decreased hippocampal inducible nitric oxide synthase (iNOS) expression in SAMP8 mice. There was no difference in iNOS expression between SAMR1 and vehicle-treated SAMP8 mice. Subchronic (5 days) administration of an iNOS inhibitor, 1400 W, shortened the immobility time in SAMP8 mice. These results suggest that NYT prevents an increase in immobility time of SAMP8 mice by decreasing iNOS levels in the hippocampus. Therefore, the antidepressive effect of NYT in older patients might be mediated, at least in part, by the downregulation of iNOS in the brain. Our data suggest that NYT is useful to prevent the onset of depression with aging.

## 1. Introduction

Depression in older adults is common, with 8.2%–41.8% of this population exhibiting depressive symptoms globally [1–5]. Severe depression, in particular, is associated with a marked reduction in the quality of life in older patients [6]. Furthermore, depression in older adults is suggested to increase the risk of frailty [7]. Therefore, therapy for depression is important for healthy living in this age group.

Ninjinyoeito (NYT) is a traditional Japanese herbal medicine (Kampo medicine) that originates in China. NYT is composed of 12 medicinal herbs (Table 1). The extract of NYT is approved in Japan and is prescribed for symptoms such as fatigue, cold limbs, anorexia, night sweats, and anemia. Recent clinical studies have demonstrated that NYT has antidepressive effects in older patients with various diseases, including chronic obstructive pulmonary disease and Alzheimer's disease [8–10]. However, the mechanisms underlying the therapeutic effectiveness of NYT for depression in older patients are unclear.

Senescence-accelerated mouse (SAM) strains established from AKR/J mice have a common genetic background [11]. Although SAM-resistant (SAMR) mice display normal aging, SAM-prone (SAMP) substrains show early onset of age-related alterations [12]. Among the SAMP substrains, SAMP8 mice exhibit age-related behavioral deficits, including cognitive dysfunction and emotional disturbances [13–15], and 4-month-old SAMP8 mice exhibit depression-like behavior [15]. Therefore, SAMP8 mice are a useful model for examining age-related depressive symptoms.

A commonly used antidepressant, fluoxetine, inhibits hippocampal inducible nitric oxide synthase (iNOS) gene expression and alleviates depression-like behavior in mice exposed to chronic mild stress [16]. The specific iNOS inhibitor *N*-(3-(aminomethyl)benzyl)acetamidine (1400 W) also attenuates stress-induced depression-like behavior [17]. These findings suggest that inactivation of iNOS is involved in the antidepressive-like effect of these drugs. Furthermore, aminoguanidine, an iNOS inhibitor, ameliorates depression-like behavior and blocks the increase in hippocampal iNOS expression [18]. Intrahippocampal administration of aminoguanidine has an antidepressive-like effect in mice subjected to chronic mild stress [19]. Thus, hippocampal iNOS may play a key role in the antidepressive action of these drugs.

In the present study, we investigate whether NYT attenuates depression-like behavior and modulates hippocampal iNOS expression in SAMP8 mice. Furthermore, we examine the effect of an iNOS inhibitor on the depression-like behavior in these mice, with the aim of clarifying the mechanisms underlying the antidepressive action of NYT in older patients.

## 2. Materials and Methods

### 2.1. Animals

All procedures involving animal care and use were carried out in accordance with the regulations stipulated by the Experimental Animal Care and Use Committee of Fukuoka University (#1714148, #1915122). Male SAMP8 and SAMR1 mice, 10 weeks of age, were purchased from Japan SLC (Hamamatsu, Japan). Mice were housed individually in cages at a temperature of 23 ± 2°C and a relative humidity of 60 ± 10% under a 12/12 h light-dark cycle (lights on: 07: 00–19: 00). Food and water were available *ad libitum*. Body weight was measured weekly to monitor health status. Sample size was determined on the basis of reports examining the effects of Kampo medicines on depression-like behavior in mice [20, 21]. This study is reported following the Animal Research: Reporting of In Vivo Experiments (ARRIVE) guidelines (Supplementary Table 1).

### 2.2. Drugs and Reagents

NYT extracts (lot. nos. 372176700 and 352223500) were gifted by Tsumura & Co. (Tokyo, Japan). The composition of the NYT extract and the details of its preparation have been previously described [22]. NYT was added to the bottled drinking water so that the mice ingested NYT at doses of 300 or 1,000 mg/kg/day (NYT300 or NYT1000) from 12 to 35 weeks of age. Bottled water was replaced weekly. Based on body weight and the amount of water that the mice drank over a week, the amount of NYT to be added to the new bottle the following week was calculated. The iNOS inhibitor 1400 W (Selleck Chemicals, Houston, TX, USA) was dissolved in saline. For SAMP8 mice, 1400 W was administered intraperitoneally once a day for 5 days. The fifth 1400 W administration was carried out 1 h before the tail suspension test.

### 2.3. Tail Suspension Test

The tail suspension test was carried out according to a previous report with modification [23]. Mice were suspended by the tail using rubber tape for 10 min. Duration of immobility was blindly measured throughout the 10 min period.

### 2.4. Elevated plus Maze Test

The procedure was based on that described by Egashira et al. [24]. The plus maze apparatus was composed of two open and two closed arms elevated to a height of 50 cm. The luminance on the open arm was about 45 lux. The time spent in the open and closed arms and the number of closed arm entries were used for analyses. The test duration was 5 min.

### 2.5. Western Blotting

Frontal cortex and hippocampus were homogenized in T-PER tissue protein extraction reagent (Thermo Fisher Scientific, Waltham, MA, USA) containing protease inhibitor cocktail and phosphatase inhibitor cocktail (Nacalai Tesque, Kyoto, Japan). After centrifugation, supernatants were separated by SDS-PAGE and transferred to polyvinylidene difluoride membranes. After blocking with blocking one reagent (Nacalai Tesque), the membranes were incubated with primary antibody (anti-iNOS antibody (18985-1-AP, Proteintech, Rosemont, IL, USA), anti-aryl hydrocarbon receptor antibody (17840-1-AP, Proteintech), anti-guanylate cyclase *β1* antibody (160897, Cayman Chemical, Ann Arbor, MI, USA), and anti-*β*-actin antibody (ab8227, Abcam, Cambridge, UK)), followed by the appropriate horseradish peroxidase-conjugated secondary antibody. The blots were visualized using the ImmunoStar LD kit (FUJIFILM Wako Pure Chemical, Osaka, Japan) using a FluorChem imaging system (Alpha Innotech, Santa Clara, CA, USA). Band densities were analyzed using ImageJ software (National Institutes of Health, Bethesda, MA, USA).

### 2.6. Experimental Procedure

NYT was ingested from 12 to 35 weeks of age. Mima et al. demonstrated that treatment with medicinal herb from 11 weeks of age ameliorates aging-induced memory declines in SAMP8 mice [25]. Therefore, NYT ingestion was initiated referring to Mima et al. [25]. Behavioral tests were carried out at 34 weeks of age to investigate the effect of NYT, and brain samples were harvested 1 week later. Another experimental mouse group was subjected to the tail suspension test at 12 weeks of age. To analyze the effects of 1400 W, 37–51-week-old SAMP8 and SAMR1 mice were used.

### 2.7. Statistical Analysis

**Figure 1:**
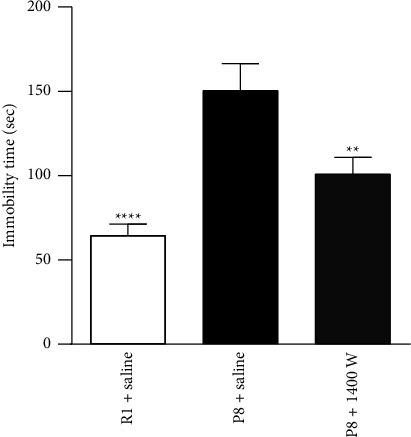
Effect of an iNOS inhibitor on depression-like behavior in SAMP8 mice. An iNOS inhibitor (1400 W; 0.375 *µ*g/kg/day) was administered for 5 days. 1400 W suppressed the increase in immobility time in P8 mice in the tail suspension test (R1 + saline: *n* = 37; P8 + saline: *n* = 27; P8 + 1400 W: *n* = 25). Values are expressed as mean ± SEM. ^*∗∗*^*p* < 0.01 and ^*∗∗∗∗*^*p* < 0.0001, compared with P8 + water, Tukey's multiple comparisons test.

**Figure 2:**
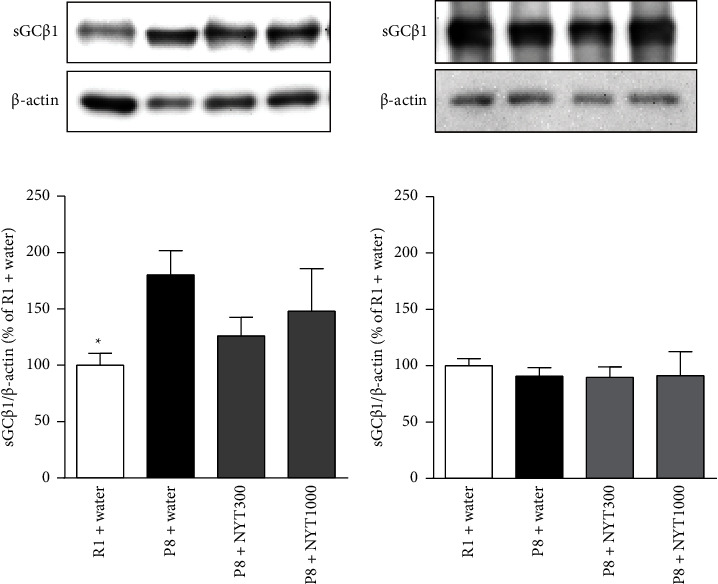
Effect of NYT on sGC*β*1 expression in the hippocampus and frontal cortex. (a) Hippocampal and (b) frontal cortical sGC*β*1 expression levels were examined by Western blot analysis. Hippocampal sGC*β*1 expression was increased in P8 mice compared with R1 mice, while NYT did not affect sGC*β*1 expression. Frontal cortical sGC*β*1 expression did not differ among the groups (R1 + water: *n* = 8; P8 + water: *n* = 8; P8 + NYT300: *n* = 7; P8 + NYT1000: *n* = 5). Values are expressed as mean ± SEM. ^*∗*^*p* < 0.05, compared with P8 + water, Dunnett's multiple comparisons test.

Data were evaluated using one-way analysis of variance followed by Tukey's or Dunnett's multiple comparisons test using Prism 6.04 software (GraphPad, La Jolla, CA, USA). Three outliers and one outlier were removed from the datasets in Figure1 (P8 + 1400 W, *n* = 3) and Figure 2(b) (P8 + NYT300, *n* = 1), respectively. Outliers were identified using the outlier identification (ROUT) method, and moreover, removed if their value was more than 2.5-fold that of the group mean. One mouse dataset (P8 + water, *n* = 1) could not be analyzed in the elevated plus maze test because the video camera did not work properly. The criterion for statistical significance was *p* < 0.05. Data are shown as mean ± standard error of the mean (SEM).

## 3. Results

### 3.1. NYT Attenuates Depression-Like Behavior in SAMP8 Mice in the Tail Suspension Test

**Figure 3:**
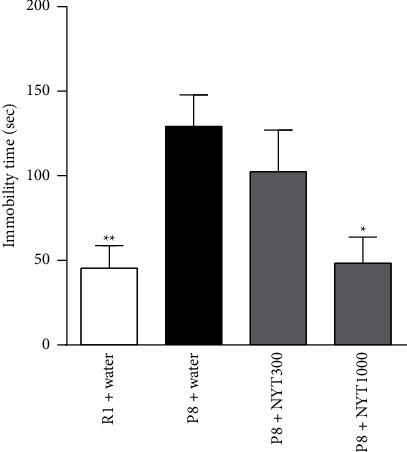
Effect of NYT on depression-like behavior in the tail suspension test. The immobility time in the tail suspension test was analyzed as an index of depression-like behavior. NYT (300 mg/kg, NYT300; 1000 mg/kg, NYT1000) was given from 12 weeks of age. The 34-week-old SAMP8 (P8) mice showed a prolonged immobility time compared with that of age-matched SAMR1 (R1) mice. NYT1000 significantly suppressed the increase in immobility time in P8 mice (R1 + water: *n* = 8; P8 + water: *n* = 8; P8 + NYT300: *n* = 8; P8 + NYT1000: *n* = 5). Values are expressed as mean ± SEM. ^*∗*^*p* < 0.05 and ^*∗∗*^*p* < 0.01, compared with P8 + water, Dunnett's multiple comparisons test.

Immobility time in the tail suspension test was used to evaluate the effect of NYT on depression-like behavior. Thirty-four-week-old SAMP8 mice showed a prolonged immobility time compared with that of age-matched SAMR1 mice (Figure 3). NYT1000 significantly shortened the immobility time in SAMP8 mice, while NYT300 had no effect. Another experiment showed immobility time of SAMP8 mice was comparable to that of SAMR1 mice at 12 weeks of age (Supplementary Figure 1), suggesting that SAMP8 mice do not show depression-like behavior at 12 weeks of age. These results suggest that NYT has an antidepression-like effect in mice with age-related depression.

**Figure 4:**
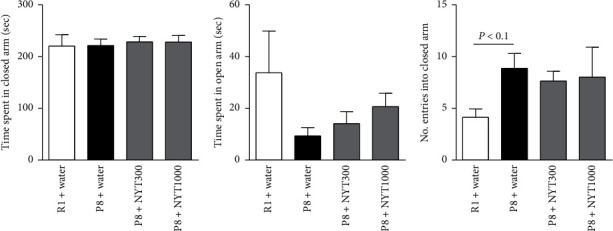
Effect of NYT on anxiety-like behavior in the elevated plus maze test: (a) the time spent and (c) the number of entries in the closed arm and (b) the time spent in the open arm of the elevated plus maze were analyzed as an index of anxiety-like behavior. All mice spent similar times in each arm. P8 mice tended to show an increase in the number of entries into the closed arm. NYT did not affect the number of entries (R1 + water: *n* = 8; P8 + water: *n* = 7; P8 + NYT300: *n* = 8; P8 + NYT1000: *n* = 5). Values are expressed as mean ± SEM. Dunnett's multiple comparisons test.

The time spent in the closed arm of the elevated plus maze did not differ among the groups, nor did the time spent in the open arm (Figures 4(a) and 4(b)), suggesting that SAMP8 mice did not exhibit anxiety-like behavior in the elevated plus maze. SAMP8 mice demonstrated a tendency toward an increase in the number of entries into the closed arm (Figure 4(c)), but the observed tendency was not significant. NYT did not affect the behavior of SAMP8 mice in this test. Considering that the time spent in each arm and the number of entries into the closed arm were not significantly different between SAMP8 and SAMR1 mice, locomotion and anxiety levels of SAMP8 mice were similar to those of SAMR1 mice. NYT did not affect spontaneous locomotion and anxiety. Therefore, the antidepression-like effect of NYT may not be implicated in spontaneous locomotion.

### 3.2. NYT Decreases iNOS Expression in the Hippocampus

**Figure 5:**
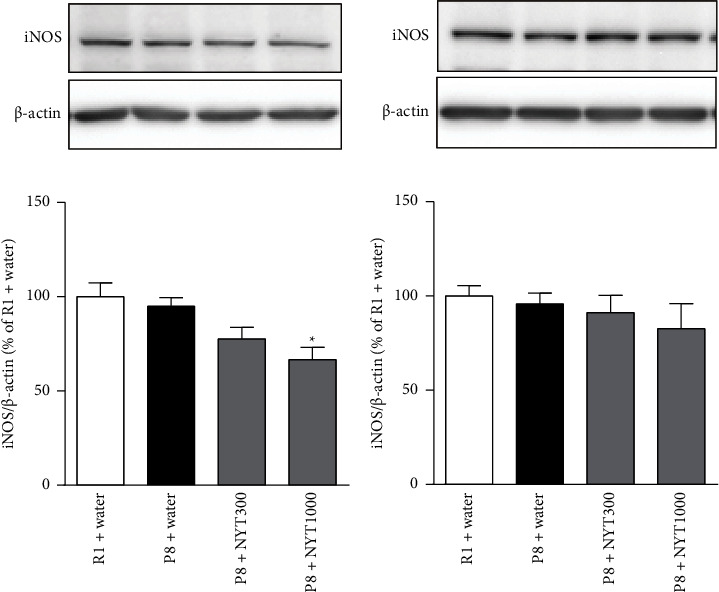
Effect of NYT on iNOS expression in the hippocampus and frontal cortex: (a) hippocampal and (b) frontal cortical iNOS expression levels were examined by Western blot analysis. Hippocampal iNOS expression was decreased by NYT1000, while frontal cortical iNOS expression was not affected (R1 + water: *n* = 8; P8 + water: *n* = 8; P8 + NYT300: *n* = 8; P8 + NYT1000: *n* = 5). Values are expressed as mean ± SEM. ^*∗*^*p* < 0.05, compared with P8 + water, Dunnett's multiple comparisons test.

The effect of NYT on iNOS expression was examined because iNOS is implicated in depression-like behavior [17, 18]. NYT1000 decreased iNOS levels in the hippocampus, but did not affect frontal cortical iNOS levels (Figure 5). There were no differences in iNOS levels between SAMP8 and SAMR1 mice in either tissue. These results suggest that NYT exerts its antidepression-like effect in SAMP8 mice, at least in part, by downregulating iNOS, although iNOS does not appear to be a direct cause of the depression-like behavior in these animals.

### 3.3. Inhibition of iNOS Ameliorates Depression-Like Behavior in SAMP8 Mice

To examine whether a reduction in iNOS activity affects depression-like behavior in SAMP8 mice, an iNOS inhibitor, 1400 W, was administered in these animals. Administration of 1400 W shortened the immobility time in SAMP8 mice (Figure 1). This result suggests that inactivation of iNOS alleviates depression-like behavior in SAMP8 mice, supporting the hypothesis that NYT attenuates depression-like behavior by downregulating iNOS.

### 3.4. NYT Tends to Decrease Aryl Hydrocarbon Receptor (AhR) Expression in the Hippocampus

**Figure 6:**
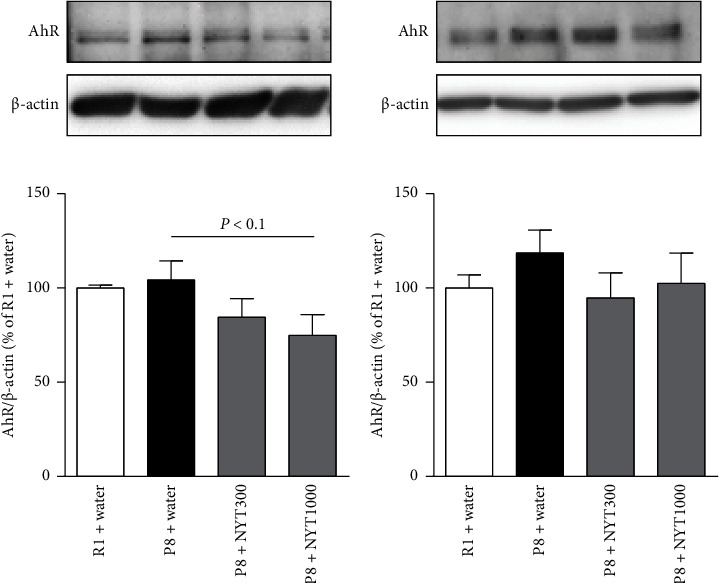
Effect of NYT on AhR expression in the hippocampus and frontal cortex. (a) Hippocampal and (b) frontal cortical AhR expression levels were examined by Western blot analysis. NYT1000 showed a tendency to reduce hippocampal AhR expression, without affecting frontal cortical AhR expression (R1 + water: *n* = 8; P8 + water: *n* = 8; P8 + NYT300: *n* = 8; P8 + NYT1000: *n* = 5). Values are expressed as mean ± SEM. Dunnett's multiple comparisons test.

AhR is a ligand-activated transcription factor [26] that modulates iNOS expression [27, 28]. Therefore, we analyzed its expression in SAMP8 and SAMR1 mice. NYT1000 tended to decrease AhR expression in the hippocampus, although AhR levels were similar in SAMP8 and SAMR1 mice (Figure 6(a)). There were no differences in frontal cortical AhR levels between SAMP8 and SAMR1 mice (Figure 6(b)). The effect of NYT on AhR expression appeared to parallel its effect on iNOS expression. Therefore, AhR may be involved in the NYT-induced reduction in iNOS levels in the hippocampus.

### 3.5. SAMP8 Mice Have Increased Soluble Guanylate Cyclase *β*1 Expression

The nitric oxide (NO)/soluble guanylate cyclase (sGC)/cyclic guanosine monophosphate (cGMP) signaling pathway is implicated in depression-like behavior [18, 29–33]. Therefore, we analyzed sGC*β*1 expression in the mice. In the hippocampus, sGC*β*1 expression was higher in SAMP8 mice than in SAMR1 mice (Figure 2(a)), suggesting that increased sGC*β*1 levels are associated with depression-like behavior. However, NYT did not affect sGC*β*1 expression. Given that NO increases sGC*β*1 activity, the NYT-induced downregulation of iNOS may decrease the activity, but not the expression of sGC*β*1 by reducing NO production.

## 4. Discussion

Kampo-hozai, a group of Kampo medicines that includes NYT [34], can make up for deficiencies in physical and mental energy. Recent clinical studies have shown that NYT ameliorates depressive symptoms in older patients [8–10]. In the present study, we examined the mechanisms underlying the antidepression-like effect of NYT using SAMP8 mice, which exhibit accelerated aging. Treatment with NYT from a young age prevented onset of depression-like behavior in these animals. An iNOS inhibitor also ameliorated the depression-like behavior. Furthermore, NYT decreased iNOS expression in the hippocampus of SAMP8 mice. These findings suggest that NYT exerts a prophylactic effect on depression-like behavior by decreasing iNOS levels.

Herbal extracts or components, which are included in NYT, are reported to affect iNOS expression. *Panax ginseng* extract has an anti-depressive effect and suppresses iNOS expression in a mouse model of stress-induced depression [35]. Catalpol, a component of *Rehmanniae radix*, also inhibits hippocampal iNOS gene expression and alleviates depression-like behavior in animal models of depression induced by chronic mild stress or chronic corticosterone administration [36, 37]. Atractylenolide III, a component of Atractylodis rhizoma, has an antidepression-like effect in rats [38] and attenuates iNOS expression in lipopolysaccharide (LPS)-treated microglia [39]. Furthermore, *Polygalae radix* extract inhibits iNOS expression in LPS-treated microglia [40]. Extract of Astragali radix and components of Rehmanniae radix (2,5-dihydroxyacetophenone), Schisandrae fructus (Schisandrin), and Glycyrrhizae radix (total flavonoids) also inhibit iNOS gene expression in a macrophage cell line [41–44]. Given that these components and extracts inhibit iNOS expression in microglia and macrophages, NYT may also suppress iNOS expression in these cells.

General antidepressants downregulate iNOS, for example, fluoxetine inhibits iNOS mRNA expression in the hippocampus of rats with stress-induced depression and in the substantia nigra of LPS-injected rats [16, 45]. Fluoxetine and paroxetine suppress iNOS mRNA expression in 1-methyl-4-phenyl-1,2,3,6-tetrahydropyridine (MPTP)-treated mice [46, 47]. These findings suggest that the antidepressant effect of selective serotonin reuptake inhibitors (SSRIs) involves the downregulation of iNOS. Tricyclic antidepressants also affect iNOS expression. For example, amitriptyline (a norepinephrine/serotonin reuptake inhibitor) and clomipramine (a serotonin reuptake inhibitor) inhibit iNOS expression in the brain, while desipramine (a norepinephrine reuptake inhibitor) does not [48]. Given that SSRIs and amitriptyline and clomipramine affect serotonergic metabolism, the serotonergic system may play a key role in the regulation of iNOS expression. We previously reported that the antidepression-like effect of NYT is inhibited by 5-HT_1A_ receptor antagonist [49]. Therefore, NYT may attenuate iNOS expression via the serotonergic system.

AhR is widely expressed in the brain, including the hippocampus [50, 51]. Neurons, microglia, astrocytes, and oligodendrocytes all express AhR [52]. AhR modulates depression-like behavior, and AhR antagonism ameliorates depression-like behavior induced by LPS [53]. Furthermore, AhR KO mice show attenuation of depression-like behavior [54]. Therefore, NYT may attenuate depression-like behavior in SAMP8 mice in part by reducing AhR expression. Indeed, AhR KO mice show inhibition of the LPS-induced increase in iNOS expression [28]. Given these observations, NYT may reduce iNOS expression by inhibiting AhR signaling, thereby ameliorating depression-like behavior.

The present study has at least two limitations. First, iNOS expression did not differ between SAMR1 and SAMP8 mice. Therefore, we did not demonstrate direct association between iNOS expression and depression-like behavior. However, administration of iNOS inhibitor to SAMP8 mice induced antidepression-like effects. The iNOS inhibitor also decreased the immobility time of normal Swiss mice in a forced swimming test, which is another test that examines the effects of antidepressants [55]. These results suggest that iNOS inhibition indirectly affects the neuropathology of depression-like behavior in SAMP8 mice. Second, we could not measure cGMP or NO levels. The NO/sGC/cGMP signaling pathway is implicated in depression-like behavior [18, 29–33]. Therefore, measuring these levels would help clarify whether NYT ameliorates depression-like behavior in mice with accelerated senescence by modulating NO and cGMP levels. It has been reported that NO levels are increased in the SAMP8 mouse brain [56, 57], while cGMP levels in the brain have not been investigated to our knowledge. sGC is a key enzyme in the NO signaling pathway. The binding of NO to sGC leads to a significant increase in cGMP [58]. The present study demonstrated an increase in sGC*β*1 levels in the hippocampus of SAMP8 mice, implying that cGMP levels may be increased in SAMP8 mice. Given that iNOS expression was not increased in SAMP8 mice, increased sGC*β*1 levels may trigger depression-like behavior of SAMP8 mice.

## 5. Conclusions

Accelerated aging is associated with depression-like behavior in mice. Chronic treatment of NYT prevented onset of depression-like behavior and decreased hippocampal iNOS expression. Our findings suggest that NYT may alleviate the onset and development of late-life depression in humans by downregulating iNOS expression in the brain.

## Figures and Tables

**Table 1:** Formula of Ninjinyoeito (NYT). The formula was used to produce 6 g of NYT dried extract.

Crude drug	Botanical origin	g
*Angelicae acutilobae radix*	Root of *Angelica acutiloba* Kitagawa	4.0
*Astragali radix*	Roots of *Astragalus membranaceus* Bunge and *Astragalus mongholicus* Bunge	1.5
*Atractylodis rhizoma*	Root of *Atractylodes macrocephala* Koidzumi	4.0
*Cinnamomi cortex*	Bark of *Cinnamomum cassia* Blume	2.5
*Citri unshiu pericarpium*	Peel of *Citrus unshiu* Marcowicz	2.0
*Ginseng radix*	Root of *Panax ginseng* C.A. Meyer	3.0
*Glycyrrhizae radix*	Root of *Glycyrrhiza uralensis* Fischer	1.0
*Paeoniae radix*	Root of *Paeonia lactiflora* Pallas	2.0
*Polygalae radix*	Root of *Polygala tenuifolia* Willdenow	2.0
*Poria*	Sclerotium of *Wolfiporia cocos* Ryvarden et Gilbertson	4.0
*Rehmanniae radix*	Root of *Rehmannia glutinosa* Liboschitz	4.0
*Schisandrae fructus*	Fruit of *Schisandra chinensis* Baillon	1.0

## Data Availability

The datasets generated for this study are available from the corresponding author upon request.

## Supplementary Materials

Supplementary Materials Supplementary Table 1: ARRIVE guidelines 2.0: author checklist. Supplementary Figure 1: immobility time of 12-week-old SAMP8 and SAMR1 mice in the tail suspension test.
