# Supplementary material for: Ninjinyoeito Prevents Onset of Depression-Like Behavior and Reduces Hippocampal iNOS Expression in Senescence-Accelerated Mouse Prone 8 Mice
Source: Evid Based Complement Alternat Med. 2023 Aug 9;2023:2151004. doi: 10.1155/2023/2151004 (PMC10432024; doi:10.1155/2023/2151004)
Supplement: Supplementary Materials — Supplementary Table 1: ARRIVE guidelines 2.0: author checklist. Supplementary Figure 1: immobility time of 12-week-old SAMP8 and SAMR1 mice in the tail suspension test. [file 2151004.f1.zip › Supplementary data TST NYT iNOS.docx]

Supporting information

**Evidence-Based Complementary and Alternative Medicine**

**Ninjinyoeito prevents onset of depression-like behavior and reduces hippocampal iNOS expression in senescence-accelerated mouse prone 8 mice**

Chise Taniguchi, Takuya Watanabe, Marika Hirata, Akinobu Hatae, Kaori Kubota, Shutaro Katsurabayashi, Katsunori Iwasaki

**Corresponding author:**

Takuya Watanabe Ph.D.

E-mail: twatanabe@fukuoka-u.ac.jp

**Supplementary figures**


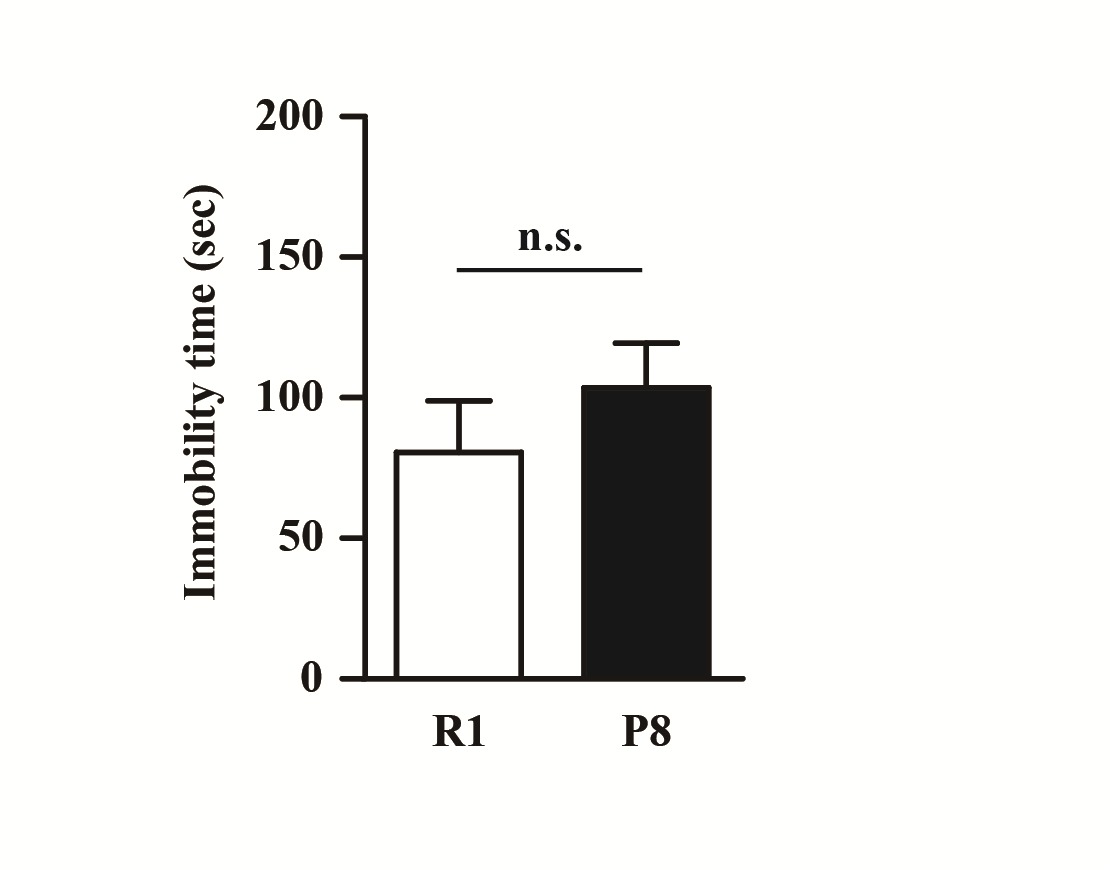


**Supplementary Fig. 1.** Immobility time of 12 week-old SAMP8 and SAMR1 mice in the tail suspension test.

SAMP8 (P8: n = 15) mice showed similar immobility time to SAMR1 (R1: n = 10) mice at 12 week-old. Values are expressed as mean ± SEM. Non-significant (n.s.), Mann-Whitney test.
